# 24-Hour Dietary Recall in the Expanded Food and Nutrition Education Program: Perspective of the Program Coordinator

**DOI:** 10.3390/nu15194147

**Published:** 2023-09-26

**Authors:** Annie J. Roe, Kavitha Sankavaram, Susan Baker, Karen Franck, Michael Puglisi, Dawn Earnesty, Teresa Henson

**Affiliations:** 1 Margaret Ritchie School of Family and Consumer Sciences, University of Idaho, Moscow, ID 83844, USA; 2 Department of Nutrition and Food Science, University of Maryland, College Park, MD 20742, USA; kavitha@umd.edu; 3 Department of Food Science and Human Nutrition, Colorado State University, Fort Collins, CO 80523, USA; susan.baker@colostate.edu; 4 Department of Family and Consumer Sciences, University of Tennessee, Knoxville, TN 37996, USA; kfranck@utk.edu; 5 Department of Nutritional Sciences, University of Connecticut, Storrs, CT 06269, USA; michael.puglisi@uconn.edu; 6 Health and Nutrition Institute, Michigan State University Extension, Saginaw, MI 48607, USA; wilcoxd4@msu.edu; 7 Family and Consumer Sciences Department, University of Arkansas at Pine Bluff, Pine Bluff, AR 71601, USA; hensont@uapb.edu

**Keywords:** 24-hour dietary recall, low-income, Expanded Food and Nutrition Education Program, group setting, peer educators

## Abstract

The purpose of this study was to determine how the 24-hour dietary recall (24HDR) is administered and how the Expanded Food and Nutrition Education Program (EFNEP) peer educators and other staff are trained on the data collection and entry process, from the EFNEP coordinators’ perspectives. This cross-sectional, quantitative study utilized an online survey to collect information from EFNEP coordinators representing 61 of 76 EFNEP programs. While 56% of the programs collected the 24HDR data starting with the first thing eaten the previous day, 49% of them started collecting data at the time of class, going backwards. Most programs, i.e., 72%, reported using a multiple-pass method; however, only one-third of them reported using the standard five-pass method. Almost all programs, i.e., 97%, reported one peer educator collecting data from a group of 2–12 clients. All programs reported collecting the 24HDR data in a group setting, with about one-third of the programs also collecting data one-on-one. Most programs, i.e., 57%, reported spending ≤4 h on the initial training of staff in how to collect 24HDR data, and 54% of them reported that the peer educators entered the data themselves. This study found that the methods used to collect answers, train the staff, and enter the 24HDR data varied across EFNEP programs and that there is a need to standardize or revise the collection of 24HDR data.

## 1. Introduction

The Expanded Food and Nutrition Education Program (EFNEP) is a federally funded community nutrition education program designed to improve the nutrition security of adults and youth from low-income households [1]. The first United States nutrition education program, EFNEP, is funded through the U.S. Department of Agriculture and National Institute of Food and Agriculture (USDA/NIFA). The program is administered by 1862 and 1890 Land-Grant Universities (LGUs) in every U.S. state, district, and territory. Faculty or staff at these institutions serve as the EFNEP coordinator to design, implement, and evaluate program activities. These coordinators provide leadership and programmatic oversight to the EFNEP at their respective institutions.

Since 1969, EFNEP classes have been taught by peer educators recruited and hired from within the communities the EFNEP serves. These positions typically require a high school diploma or a General Educational Development (GED) certificate, with additional training provided by EFNEP coordinators and supervisors. EFNEP supervisors work with peer educators and community partners to recruit adults and youth for participation in EFNEP classes.

EFNEP began as a program that provided one-on-one nutrition education lessons in the home setting. Over the years, through strong community partnerships, this model has evolved to offer similar classes in a group setting. More recently, in response to the COVID-19 pandemic, there has been an increase in virtual group classes within the EFNEP. Both of these changes have allowed for increased EFNEP reach, and each delivery method comes with challenges and nuances. For the purposes of this study, the focus was on in-person program delivery and assessment.

EFNEP history includes a rigorous evaluation of adult programs, including utilizing a validated pre-/post-questionnaire to assess behavioral changes related to diet quality, physical activity, food safety, and food resource management [2]. In addition to the questionnaire, diet quality is evaluated through the use of 24-hour dietary recall (24HDR) assessments. Adult participants complete a 24HDR before and after the EFNEP lesson series, which is used to assess changes in diet quality as a result of participation in an EFNEP class series. The programs may also use the baseline 24HDR to help guide the program content and address identified areas of improvement throughout the lessons. The 24HDR is one of the most widely used tools to assess dietary intake and, when properly administered, can be an effective and accurate method [3,4,5,6,7]. The USDA five-step multi-pass method is one of the most comprehensive and effective methods of conducting a 24HDR [8,9,10]. While the standard protocol for collecting 24HDR data includes the administration of the questionnaire by a registered dietitian, research has shown that well-trained EFNEP peer educators may collect similar quality data as compared to registered dietitians [11]. However, previous research assessing the use of 24HDR methods in EFNEP identified a need for the standardization of protocols and forms as well as for further studies to validate the use of 24HDR in group settings [12,13]. Changes in program delivery strategies as well as changes in EFNEP coordinator leadership over the last decade may have influenced the current 24HDR collection and training methods used by programs. Data from the 24HDR are analyzed and used to report EFNEP diet quality outcomes and impacts at the local, state, and national level. Having standard procedures and maintaining fidelity of data collection is critical to accurately report these types of collective impacts [14].

The purpose of this study was to determine how the 24HDR dietary assessment is delivered and how EFNEP peer educators and other staff are trained on the collection and data entry process from the perspective of EFNEP coordinators.

## 2. Materials and Methods

This cross-sectional study was part of a larger mixed methods study designed to assess the 24HDR process, including training, material utilized, and perspectives of coordinators and peer educators. This quantitative study utilized an online survey to collect information from EFNEP coordinators. The survey was designed to examine the current 24HDR collection and training methods used in the EFNEP nationwide.

### 2.1. Study Design, Participants, and Procedure

The sample population consisted of 76 EFNEP coordinators. Potential participants were contacted by email via a national EFNEP listserv requesting their participation in an online survey. The survey consisted of questions related to coordinators’ knowledge, the process, training, and implementation of the 24HDR in their EFNEP delivery. At the beginning of the web-based survey, the coordinators were asked to provide informed consent for participation in the study. The questions in the survey were based on a previous study [12]; however, they were further expanded to address current programmatic needs (increase in staff turnover and program delivery strategies). Three EFNEP coordinators reviewed the survey for content, clarity, and time requirement for completion. Since the survey questions were adapted from a previous version that was pilot-tested with EFNEP staff and nutrition professionals, no further testing was conducted to establish content and face validity. This study was approved by the University of Maryland, College Park Institutional Review Board (1416811-1).

### 2.2. Measures

The survey was administered through the Qualtrics survey creation and distribution platform and consisted of 38 closed and open-ended questions. The survey included general questions about the program characteristics, such as the education level of the peer educators delivering EFNEP lessons and the funding tier of the program. Specific survey questions captured details of the 24HDR process, including the period of the 24HDR collection (previous day or previous 24 h), the methods used to collect the 24HDR data (single-pass or multiple-pass), the data collection setting (one-on-one or group), and the group size. Other questions addressed the training of the peer educators on the 24HDR collection, the frequency of the training, the duration of the training, and the data entry procedures.

### 2.3. Data Analysis

Quantitative data from the survey were compiled and measured using descriptive statistics and are presented as frequency percentages. The analysis for this paper was generated using Qualtrics software (Qualtrics, Provo, UT, USA), Version (September 2021) of Qualtrics. Copyright^©^ (2023) Qualtrics.

## 3. Results

### 3.1. Program Characteristics

Sixty-one (of seventy-six) program coordinators completed the survey. The program characteristics are summarized in Table 1. Programs from all funding tiers were represented and included programs with peer educators whose highest level of education was a high school diploma or a GED certificate, as well as programs with peer educators with a higher educational attainment. For those programs that taught in languages other than English, Spanish was the most frequently reported language.

### 3.2. Twenty-Four-Hour Dietary Recall Process

The reported characteristics of the 24HDR process are summarized in Table 2. There was a fairly even split between programs collecting the 24HDR starting with the first thing eaten yesterday versus those starting at the time of class and going backwards. Nine programs selected multiple options and indicated that the timeframe for recall was not standardized. Most programs reported using a multiple-pass method, with more programs conducting 2–4 passes than five passes. All programs reported collecting 24-hour recall data in a group setting, with about one-third of the programs also collecting these data in a one-on-one setting. The majority of the programs (61%) always or sometimes provided the “One-Day Food Recall Summary” form to the participants after the 24-hour dietary recall data had been entered into WebNEERS for analysis.

The details of the 24HDR process when carried out in a one-on-one setting are summarized in Table 3. Regardless of the number of passes used to collect the data, the programs reported that peer educators recorded intakes more often than the clients. Probing questions were used after all foods/beverages were recorded for the 24 h period and often also after all foods/beverages were recorded for each meal or snack.

The details of the 24HDR process when conducted in a group setting are summarized in Table 4. While many programs reported collecting data with variable numbers of peer educators and clients, almost all programs reported collecting data with one peer educator and 2–12 clients. The majority of the programs reported utilizing multiple passes to collect 24HDR data. However, only one-third of them reported using the standard five-pass method.

### 3.3. Staff Training on the 24-Hour Dietary Recall

The characteristics of the staff training program in the 24HDR process are summarized in Table 5. The majority of the programs reported using “The 24-hour Food Recall, An Essential Tool in Nutrition Education, In-Service Training Program” from the Oklahoma State University Extension Service to train staff. Most programs train less than 4 h in a group setting. If a refresher training is offered (about one-third of the programs do not offer refresher training), it is offered every year. Most programs reported using a variety of training methods, including lecture, practice recalls, and props during training. There was variability reported in who provided the training, with several programs reporting a number of different individuals. The most commonly reported trainer was the LGU EFNEP coordinator or another EFNEP professional. The majority of the programs reported observing peer educators one or two times a year.

### 3.4. Data Entry Process and Training

The process for entering the 24HDR data and the training on data entry is summarized in Table 6. About half of the programs reported that peer educators entered the data they collected themselves. This is reflected in the number of people who entered the data, with the majority of programs reporting one to five people entering the data. Training on data entry typically lasted less than 8 h, with a little over half of the programs reporting training for less than 4 h. The majority of the programs reported including practice entering data into WebNEERS as part of the training program. Most programs reported not providing refresher training or only providing training if it was warranted due to data errors or changes coming from the National EFNEP office.

## 4. Discussion

This descriptive study surveyed EFNEP coordinators from 61 programs to capture how 24HDR data are collected and entered as well as how peer educators and other staff are trained on these processes. Programs from all funding tiers were represented. Program funding has the potential to impact the 24HDR process through resources allocated to such factors as materials, training, and in-person observations. Although, according to the EFNEP model, a high school diploma or a GED certificate is required for peer educators, only 31% of the program coordinators responded that 61–100% of their peer educators had a high school diploma as their highest level of education. This is important to note because, although a GED certificate is sufficient, individuals in these positions often have higher educational attainment. There is also the possibility that peer educators take advantage of educational opportunities as university employees and continue their education while working for the EFNEP. These individuals have the ability to accurately collect 24HDR data in a one-on-one setting, when adequately trained [11]. This study found that the methods used to collect the 24HDR data and the training requirements varied across LGUs administering the EFNEP. This is a real concern, as the data from the 24HDR are utilized in the EFNEP outcome reports at the local and national level. The potential for inconsistent data may be decreased by implementing best practices at the national level.

The USDA five-pass method is regarded as the gold standard in collecting 24HDR data. The five steps include (1) collecting a quick list of all foods and beverages consumed in the previous 24 h, (2) probing for forgotten foods, (3) assigning a time and eating occasion to each item consumed, (4) providing a detailed description of the portion size or amount of food eaten and any additions (such as sauces, toppings, etc.), and (5) finally probing for any other food or beverage consumed [9]. The most commonly used training tool reported by EFNEP coordinators was “The 24-hour Food Recall, An Essential Tool in Nutrition Education, In-Service Training Program.” This training protocol teaches the USDA multi-pass five-step method [15]. Not surprisingly, most EFNEP coordinators in the current study reported using a multi-pass method to collect 24HDR data. However, more programs reported conducting 2–4 passes rather than the standard five passes. While several programs reported using probing questions, it was not clear what specific steps were included in the 2–4 passes, indicating a need for a further understanding of what is feasible and what is the best practice in this setting. Previous research assessing the group 24HDR with EFNEP participants utilized a three-step method [16]. Additional variation in the 24HDR process was identified in the timeframe in which the 24HDR was carried out. As regards the starting time of the 24HDR data collection, the programs were split between starting with the first item eaten yesterday and starting at the time of the class and working backwards. The standard time frame for the 24HDR is midnight to midnight [17,18]. All variations in the protocol may influence the accuracy of the data collected and should be standardized and validated in a group setting. Part of this validation should include the number of 24HDR needed to accurately assess the dietary intake of EFNEP adult participants. While only one pre/post 24HDR is administered in the EFNEP, previous research showed that more than one 24HDR is needed to accurately estimate the usual intakes [8,19,20]. Administering more than one pre/post 24HDR may not be feasible, as it may cause undue burden to the EFNEP participants and take time away from the EFNEP lessons. A recently published qualitative study provided insights into the EFNEP 24HDR from the perspective of the EFNEP participants [21]. These perspectives should be taken into consideration when designing future standard EFNEP 24HDR protocols and training programs.

Most peer educators received less than 4 h of training on the 24HDR procedures in a group setting. It is not clear if this is adequate time for training peer educators. In the creation of a standardized 24HDR training, it would be important to identify specific knowledge and skill competencies that must be acquired, regardless of the number of training hours. Additionally, validating the process of obtaining 24HDR is necessary to provide evidence-based strategies for data collection. The current training is based on procedures that are validated for peer educators in one-on-one settings [11]; these methods may need to be adjusted for the group setting. Since collecting 24HDR has not been validated in a group setting, additional research, training, and resources are needed to optimize this process. Similarly, training on data entry was variable across the LGUs implementing the EFNEP, with a little over half of the programs reporting training for less than 4 h. Currently, there is not a standard protocol LGUs should follow when entering EFNEP 24HDR data. About half of the EFNEP coordinators reported that peer educators entered the data they collected themselves. Recent research assessing between- and within-coder reliability in one LGU administering the EFNEP concluded that while there was potential for reliable data coding and entry by the EFNEP staff, future training should be standardized [22]. Further insights into challenges and best practices in data entry may be identified through research assessing perspectives and experiences of the EFNEP peer educators themselves. Accuracy is further diminished by limitations of the data entry system, which lacks many cultural foods consumed by the EFNEP participants [13]. Additionally, there is a need to assess the benefits of the data obtained from 24HDR weighed against the cost of EFNEP staff time, the time away from lesson content, and the participant burden of filling out paperwork.

As the EFNEP has evolved, so have the evaluation tools. The EFNEP policies include recent technology guidelines [23] to allow the collection of 24HDR data through the Automated Self-Administered 24-hour (ASA24) Dietary Assessment Tool [24]. This tool has been used to assess the dietary intake of individuals with low-income [25], and standardized training tools for the EFNEP have been developed [26]. However, this tool requires internet access and the use of electronic devices to collect data, resources that may not be readily available to all EFNEP audiences. Other tools that have been updated include a validated pre/post questionnaire which is also completed by adult participants and assesses diet quality [2]. Research to compare the outcomes assessed by the EFNEP Adult Questionnaire to those assessed by the 24HDR has not been conducted. This comparison would provide important data to support the need to continue collecting 24HDR data and work to standardize the training or justify a change in EFNEP policies to reduce paperwork and maximize the teaching time in EFNEP classes.

## 5. Conclusions

The 24HDR is an efficient and effective tool in assessing dietary intake in a one-on-one setting. This tool is used in a group setting to report changes in diet quality as a result of participation in EFNEP classes. As EFNEP has evolved, so have the available evaluation tools. Additional research needs to be conducted to determine the benefits of continuing the use of 24HDR in the EFNEP. If the use of this tool is to be continued, standard forms, protocols, and training programs need to be created. These standardizations should take into account the perspectives of program coordinators, peer educators, and EFNEP participants.

## Figures and Tables

**Table 1:** Program characteristics.

Variable	Frequency (%)
Funding tier	
1 (USD 2.5 million–USD 4.4 million)	8
2 (USD 1.8 million–USD 2.5 million)	13
3 (USD 1.2 million–USD 1.8 million)	15
4 (USD 900 thousand–USD 1.2 million)	13
5 (USD 450 thousand–USD 900 thousand)	13
6 (USD 250 thousand–USD 450 thousand)	16
7 (USD 100 thousand–USD 250 thousand)	21
Peer educators’ highest education is high school diploma/GED	
0–20%	26
21–40%	25
41–60%	16
61–80%	7
81–100%	23
No response	3
Languages of programming other than English *	
Spanish	61
Chinese	3
Somali	3
Nepalese	2
Other ^1^	15
English Only	33
No response	3

* Subjects could select multiple languages, making the total frequency percent over 100. ^1^ Other languages included Russian, Chuukese, Pohnpeian, Swahili, Marshallese, Khmer, Samoan, Haitian Creole, Arabic, Mandarin, and Vietnamese.

**Table 2:** Overview of 24-hour dietary recall process.

Variable	Frequency (%)
Time Period *	
Starting with the first thing eaten yesterday	56
Starting at the time of class and going backwards in time	49
Recall period not standardized	15
Method *	
Single-pass	23
Multiple-pass with 2–4 passes	41
Multiple-pass with five passes	31
Recall method not standardized	7
Other ^1^	3
Setting *	
One-on-one	34
Group	100
Volunteers involved in collection	16
After Recall *	
Participants always received the “One-Day Food Recall Summary” form	18
Participants sometimes received the “One-Day Food Recall Summary” form	43
Participants never received the “One-Day Food Recall Summary” form	31
Unsure if participants received the “One-Day Food Recall Summary” form	8

* Subjects could select multiple answers, making the total frequency percent over 100. ^1^ Other methods described variations due to COVID-19, e.g., in various situations, especially during the COVID-19 pandemic, it was possible that not all 5 passes were implemented, due to time or communication constraints. During the COVID-19 pandemic, we also adapted the 5-pass method to a Qualtrics survey; so, more participants completed the survey on their own as opposed to group or one-on-one collection.

**Table 3:** One-on-one 24-hour dietary recall process.

Variable	Frequency (%)
Method *	
Single-pass, educator records intake	33
Single-pass, client records intake	10
Multiple-pass with 2–4 passes, educator records intake	33
Multiple-pass with 2–4 passes, client records intake	14
Multiple-pass with five passes, educator records intake	24
Multiple-pass with five passes, client records intake	19
Other ^1^	5
Probing Questions *	
After all foods/beverages were recorded for the 24 h period	38
After all foods/beverages were recorded for each meal or snack	5
Both	57
Recall method not standardized	5
I do not know	5

* Subjects could select multiple answers, making the total frequency percent over 100. ^1^ Paraprofessional then translated the answers from Spanish to English for data entry.

**Table 4:** Group 24-hour dietary recall process.

Variable	Frequency (%)
Setting *	
One peer educator and 2–12 clients	97
One peer educator and 13–20 clients	44
Two peer educators and 13–20 clients	16
One peer educator and more than 20 clients	15
Two peer educators and more than 20 clients	7
Method *	
Single-pass, client records intake	25
Multiple-pass with 2–4 passes, client records intake	41
Multiple-pass with five passes, client records intake	33

* Subjects could select multiple answers, making the total frequency percent over 100.

**Table 5:** Overview of staff training on the 24-hour dietary recall process.

Variable	Frequency (%)
Training Program *	
The 24-hour Food Recall, An Essential Tool in Nutrition Education, In-Service Training Program—Oklahoma State University Extension Service	62
Eat Smart—Louisiana State University	5
Nutrition Education Process Manual—Rutgers University, New Jersey	2
Navigating for Success—Cornell University, New York	8
Cent$ible Nutrition Program Initial Training Manual, University of Wyoming	2
UC Davis Food Tracker	8
Other ^1^	28
No response	3
Duration of Initial Training	
>4 h	64
<4 h, ≤8 h	23
>8 h	10
No Response	3
Refresher Training	
No refresher training	33
6 months	2
Annually	33
2 years	10
Other ^2^	20
No Response	3
Training Methods and Materials *	
Lecture	64
DVD	30
Other recordings	20
Practice recalls taken	75
Props (cups, plates, etc.) available during training	75
Peer educators are trained in a group setting	80
Peer educators are trained in a one-on-one setting	38
Varies	3
Other ^3^	8
No Response	2
Training Provider *	
EFNEP coordinator	67
Regional EFNEP supervisors	43
County Family and Consumer Sciences faculty	13
LGU Extension Specialists	23
Other ^4^	10
LGU EFNEP professional	52
No Response	3
Observation Frequency	
At least once per each lesson series	5
Once in 6 months per each peer educator	30
Once in every year per each peer educator	36
Other ^5^	16
I do not know	10
No Response	3
Food Models/Props Used *	
Props/aids (e.g., different sizes of cups, plates, deck of cards, measuring cups)	84
Food models	77
Posters/charts	54
No props/aids	5
Other ^6^	7
No Response	5

* Subjects could select multiple answers, making the total frequency percent over 100. ^1^ Other training programs included teach-back, WebNEERS review, review as lessons in ESBA and Today’s Mom curriculum, Families Eating Smart Moving More, training providing by the coordinator, training by others who had been trained, instructions for 5-pass method developed in-house, one-to-one observations, mentoring, shadowing. ^2^ Other refresher training responses included, discussed in each workshop; plan for more consistent training; refresher training when curriculum was updated; reviewed for online delivery and phone collection; often cut from annual training list; refresher offered based on supervisor recommendations; refresh if educators had questions; no formal plan; offer as needed. ^3^ Other training methods included written handouts included in new employee training manual. In total, our educators went through the OSU 24-hour recall training video with their area coordinator, participated in up to two ASA24 recalls as participants, shadowed other educators in their region, shadowed the administrative assistants that entered the recall data into WebNEERS, and then conducted mock recalls with other educators in their region. Watching a support staff person try to enter data into WebNEERS helped staff see how specific they needed to be. We “blinded” actual food recalls from our program and had our paraprofessional educators evaluate them for completeness and tell us what the “mystery educator” might have failed to do in collecting that recall. ^4^ Other training providers listed included peer-to-peer providers; a trainer; the director; the annual conference for training and professional development by internal and external speakers; the state EFNEP program leader; the state EFNEP assistant director. ^5^ Other observation frequency responses included random, depends if teaching observations hit the first lesson; once or twice a year, but not consistent; recommended at least once per year; 4 times; 2–3 visits per year but not all falling on a recall; as needed; almost monthly with remote program delivery; at least 4 times per year; annual, not all include a recall. ^6^ Other responses to Food Models/Props used included handouts with forgotten foods; do not know; videos that accompanied the NC State Eating Smart Moving More curriculum.

**Table 6:** Overview of the data entry process for the 24-hour dietary recall.

Variable	Frequency (%)
Who Enters Data *	
Each paraprofessional educator enters the data the educator collected	54
Support staff at the local (county) level	16
Support staff at the regional (multi-county) level	11
Support staff at the state (university) level	34
Other ^1^	8
Number of People Who Enter Data	
1	15
2–3	33
4–5	21
6–10	11
11–20	8
>20	8
Varies ^2^	2
No response	2
Training Time	
<4 h	57
>4 h, ≤8 h	21
>8 h	10
Varies ^3^	2
Do not train	7
No response	3
Refresher Training	
No refresher training	41
6 months	3
Annual	16
2 years	7
Varies ^4^	20
Other ^5^	10
No response	3
Training Methods *	
Lecture	39
Practice entering data into WebNEERS	82
Staff trained in a group setting	46
Staff trained in a one-on-one setting	61
No specific training	3
Other ^6^	8
No response	3

* Subjects could select multiple answers, making the total frequency percent over 100. ^1^ Other responses to data entry staff included student employees at the state level; EFNEP coordinator; ASA24 automatically imports; paraprofessional beginning the data entry, and data entry finished and corrected at the state level as needed. ^2^ Other responses to the number of people who entered data included entering paper in-person food recalls by 14 different paraprofessional staff and ASA24 imports by 2 people only. ^3^ Comments on data entry time included those data entry personnel who entered adult data shadow trained for several days, but not every data entry specialist entered adult data. ^4^ Comments on refresher training variability included depending on the educator; depending on the equality of data during checks; only if NIFA had updated rules/concerns; as needed. ^5^ Other refresher training comments included no refresher trainings, but continuous assistance with data entry and learning what to do; do refresher if data look incorrect or implausible; troubleshoot as needed; as needed. ^6^ Other data entry training methods included training by the data manager; in-house guides developed for troubleshooting common problems; sending videos when options changed in WebNEERS or policies changed; lecture really just going over written directions.

## Data Availability

The data presented in this study are available on request from the corresponding author.
